# The pulsing brain: state of the art and an interdisciplinary perspective

**DOI:** 10.1098/rsfs.2024.0058

**Published:** 2025-04-04

**Authors:** Andrea Lecchini-Visintini, Jacobus J. M. Zwanenburg, Qiuting Wen, Jennifer K. Nicholls, Thomas Desmidt, Stefan Catheline, Jatinder S. Minhas, Chiara Robba, Mariia Dvoriashyna, Alexandra Vallet, Jeffrey Bamber, Mehmet Kurt, Emma M. L. Chung, Samantha Holdsworth, Stephen J. Payne

**Affiliations:** ^1^ School of Electronics and Computer Science, University of Southampton, Southampton, UK; ^2^ Translational Neuroimaging Group, Center for Image Sciences, UMC Utrecht, Utrecht, The Netherlands; ^3^ Department of Radiology and Imaging Sciences, Indiana University School of Medicine, Indianapolis, IN, USA; ^4^ Weldon School of Biomedical Engineering, Purdue University, West Lafayette, IN, USA; ^5^ Department of Cardiovascular Sciences, Cerebral Haemodynamics in Ageing and Stroke Medicine (CHiASM) Research Group, University of Leicester, Leicester, UK; ^6^ University Hospitals of Leicester NHS Trust, Leicester, UK; ^7^ CHRU de Tours, Tours, France; ^8^ University of Lyon, Lyon, Auvergne-Rhône-Alpes, France; ^9^ Department of Surgical Sciences and Integrated Diagnosis, University of Genoa, Genova, Italy; ^10^ IRCCS Policlinico San Martino, Genova, Italy; ^11^ School of Mathematics and Maxwell Institute for Mathematical Sciences, University of Edinburgh, Edinburgh, UK; ^12^ Ecole nationale supérieure des Mines de Saint-Étienne, INSERM U 1059 Sainbiose, Saint-Étienne, France; ^13^ Institute of Cancer Research, London, UK; ^14^ Royal Marsden NHS Foundation Trust, London, UK; ^15^ Department of Mechanical Engineering, University of Washington, Seattle, WA, USA; ^16^ School of Life Course and Population Sciences, King's College London, London, UK; ^17^ Mātai Medical Research Institute, Tairāwhiti-Gisborne, New Zealand; ^18^ Faculty of Medical and Health Sciences & Centre for Brain Research, University of Auckland, Auckland, New Zealand; ^19^ Institute of Applied Mechanics, National Taiwan University, Taipei, Taiwan

**Keywords:** brain tissue pulsation, magnetic resonance imaging medical diagnostics, ultrasound medical diagnostics, mathematical modelling of biological tissues, mathematical modelling of biological fluids

## Abstract

Understanding the pulsing dynamics of tissue and fluids in the intracranial environment is an evolving research theme aimed at gaining new insights into brain physiology and disease progression. This article provides an overview of related research in magnetic resonance imaging, ultrasound medical diagnostics and mathematical modelling of biological tissues and fluids. It highlights recent developments, illustrates current research goals and emphasizes the importance of collaboration between these fields.

## 1. Introduction

Brain tissue is known to mechanically pulsate, with the cardiac cycle being the most evident forcing factor. This phenomenon has been recognized since antiquity, with early descriptions derived from examinations of cranial wounds and reported in medical texts from ancient Egypt [1]. In new-borns, these pulsations can be observed through the anterior fontanelle. However, as the cranial sutures close and the skull becomes rigid, detecting brain pulsations becomes a significant technical challenge. Brain tissue pulsations are intrinsically linked to the motion of neurofluids, which, in the broader sense, include all fluids in the dural envelope, such as blood, cerebrospinal fluid (CSF) and interstitial fluids (ISF) [2]. Recent technological advancements have enabled reliable recordings of brain pulsations, unlocking research that offers new perspectives on brain health and disease progression. This may open potential clinical diagnostic and therapeutic novelties, as the pulsation of the brain is strictly related to its physiology.

The aim of this perspective article is to highlight current research on the recent developments in magnetic resonance imaging (MRI) and ultrasound technologies used to capture the motion of brain tissue and neurofluids. These technologies provide direct measurements of displacement, are non-invasive and pose minimal ethical concerns, making them highly accessible for research studies. The article also focuses on parallel developments in mathematical models describing these phenomena.

Section 2 illustrates MRI technology. The section highlights how decades of research are now reflected in the integration of tissue and fluid motion acquisition capabilities into commercial MRI platforms. This part explores multiple active research threads and outlines the pathway from the development of imaging markers to their application in clinical research.

Section 3 focuses on ultrasound technology. Ultrasound recordings of brain pulsation have been in continuous development since the 1950s [3]. Current research has led to functioning prototypes for the acquisition of tissue pulsation, enabling cost-effective clinical studies, which however are not yet available commercially. The presentation emphasizes portability, low cost and ease of availability, as the key advantages of this technology, and illustrates current limitations and research goals.

Section 4 illustrates current research on modelling brain tissue and neurofluid motions, as well as the integration of data in the models. The modelling of the pulsing brain is still in its early stages, but notable successful initiatives, such as the Living Heart Project [4], the Physiome Project [5] and the Virtual Physiological Human (www.vph-institute.org), have demonstrated how the development and validation of *in silico* models can drive progress in clinical practice, device development and validation.

This article focuses on the technological advancements and the mathematical modelling aspects. However, it will also highlight how these developments unlock insights into the physiology of the brain based on understanding the interaction between tissue movement and the flows of blood, CSF and ISF. Examples of clinically relevant investigations focused on brain pulsation include idiopathic syringomyelia and Chiari malformation [6,7], hydrocephalus [8], aneurysm [9], cerebral microvascular damage [10–12], white matter abnormalities [13,14], ageing and neurodegenerative diseases [15–21], depression [22,23], haemorrhage and oedema [24–26]. In the case of neurodegenerative diseases, the prevailing hypothesis is that increased blood pulse pressure stresses surrounding tissues, weakening the blood–brain barrier and disrupting neuronal chemical balance [27,28]. Despite these associations, a direct cause-and-effect relationship between blood pulsatility and brain tissue damage is not established [29], and it remains uncertain if targeting pulsatility itself could prevent such issues.

However, brain pulsations are not always harmful. On the contrary, they appear to be necessary for brain health. Although there is some disagreement on several aspects, it is generally agreed that arterial pulsations are an important motive force of the glymphatic system, a recently discovered brain clearance mechanism [30–32]. The glymphatic system relies on the flow of CSF through a network of perivascular channels along blood vessels and its exchange with ISF, where metabolic wastes are produced before it exits the brain. Cerebral vessel pulsatility, notably due to cardiac contractions, facilitates oscillatory CSF flow and enhances glymphatic clearance. In animal models, solutes stagnate when these pulsations are reduced, either through artificial vascular ligation [33] or in conditions such as ageing [34] and acute hypertension [35]. Lower frequency pulsations, such as vasomotion often linked to neuronal activity, also support CSF flow [36,37]. The specific mechanisms through which vascular pulsation enhances transport in the brain are still debated; some propose it creates a net flow [38,39], while others suggest it primarily enhances mixing within the CSF [40,41]. Understanding the relationship between brain pulsatility and the glymphatic system is important as it is implicated in several factors of brain health such as regulating nutrient supply, signalling, waste elimination and drug delivery.

The technological advancements and mathematical modelling tools discussed in this perspective article will help advance the understanding of these processes in both health and disease.

Throughout the article, some technical terms related to material elastic deformation under the effect of forces will be used. Stress denotes force applied per unit area, and shear stress denotes force applied tangentially per unit area, both measured in pascals (Pa). Strain denotes relative displacement in the direction of the applied force. It is a relative dimensionless quantity. In a similar way, shear strain denotes relative longitudinal displacement between the parallel layers of material. Youngs’ modulus is the ratio of stress to strain and measures the stiffness of a material in the direction of the applied force. Shear modulus is the ratio of shear stress to shear strain and measures a material’s stiffness under shear deformation. Bulk modulus is the ratio between pressure and relative change in volume and measures resistance to compression. These moduli are all measured in Pa since they represent a ratio between pressure and relative dimensionless quantities. Finally, the Poisson ratio denotes the ratio of strain in the direction perpendicular to the applied force to strain in the direction of the applied force and is a relative dimensionless quantity, which measures how a material deforms in the direction perpendicular to the applied force.

## 2. Magnetic resonance imaging studies

This section explores advanced MRI methods that enable the capture of the moving brain and the dynamic fluids that influence its motion, particularly focusing on the brain tissue, blood flow and CSF, highlighting their respective capabilities and limitations. Additionally, it addresses the challenges in developing accurate and biologically specific measurement techniques, especially for capturing the complexity of brain pulsatility.

MRI sequences, such as balanced steady-state free precession (bSSFP) and phase-contrast MRI (PC-MRI), play a pivotal role in visualizing and quantifying these brain dynamics. These methods allow researchers to track fluid motion, tissue displacement and strain, which are important for understanding how blood flow and CSF movement interact with the brain tissue. As detailed in table 1, several MRI vendors offer various sequences for these purposes. The availability of these sequences across multiple MRI platforms enhances the versatility of brain motion studies, providing researchers with consistent, high-quality imaging tools.

**Table 1.** MRI techniques for measuring pulsatile brain and CSF motion: vendor-specific names, parameters and use cases.

vendor-specific name	generic name	quantitative or semi-quantitative?	use case	physiological parameters needed	commercially available?
FIESTA (GE Healthcare), TrueFISP (Siemens, Hitachi), Balanced FFE (Philips, United Imaging), Balanced SSFP (Canon), True SSFP (Toshiba)	(Cine) balanced SSFP (bSSFP)	semi-quantitative	brain tissue, CSF and/or membrane motion over the cardiac cycle [6,42]	cardiac gating. Optional: respiratory gating	yes
FIESTA-C (GE Healthcare), TrueFISP Real-Time (Siemens), Balanced Real-Time FFE (Philips, United Imaging), Balanced Real-Time SSFP (Canon)	real-time balanced SSFP (bSSFP)	semi-quantitative	real-time CSF flow, dynamic brain tissue motion [26]	no physiological gating required. Optional: cardiac logging (and respiratory depending on application)	most
2D PC-MRI (GE, Siemens, Philips, Canon, Hitachi, United Imaging)	(Cine) phase-contrast MRI	quantitative (velocity, flow rate, cm s^−1^)	brain tissue, ventricular/cervical CSF flow (e.g. at C2/C3 level of the spinal canal or at the aqueduct) and arterial and venous blood flow over the cardiac cycle in both large and small (<1 mm) vessels [43–49]	cardiac gating. Optional: respiratory gating	yes
real-time 2D PC-MRI (GE, Siemens, Philips)	real-time phase contrast MRI	quantitative (velocity, flow rate, cm s^−1^)	real-time measurement of ventricular/cervical CSF flow (e.g. at C2/C3 level of the spinal canal or at the aqueduct) and real-time arterial and venous blood flow [50–55]	no physiological gating required. Optional: cardiac logging (and respiratory depending on application)	on some vendors
4D Flow MRI (GE, Siemens, Philips, Canon, Hitachi, United Imaging Healthcare)	4D flow (extension of phase-contrast MRI that provides 3D imaging of flow over time)	quantitative (velocity, flow rate)	arterial and venous blood flow, CSF flow over the cardiac cycle [17,29]	cardiac gating. Optional: respiratory gating	yes
—	velocity-selective arterial spin labelling (VS-ASL)	quantitative (CBF, cm s^−1^, perfusion map)	selective labelling of arterial blood based on velocity rather than spatial localization, enabling perfusion imaging without requiring bolus arrival time modelling [56–58]	velocity-selective preparation module to label arterial blood, post-labelling delay, background suppression	no (research prototype)
—	cardiac-resolved vascular space occupancy (VASO) MRI	semi-quantitative (CBV-weighted signal changes)	blood-volume-weighted fMRI for detecting CBV changes in response to cardiac pulsations [59]	cardiac and respiratory logging optional, inversion recovery pulse to nullify blood signal	no (research prototype)
DENSE-MRI (GE, Siemens, Philips, Canon)	DENSE-MRI	quantitative (strain, displacement)	brain tissue deformation over the cardiac cycle [60,61,62]	cardiac gating, or cardiac/respiratory logging	on some vendors
phase-sensitive reconstruction of diffusion tensor data	SCIMI MRI (GE)	quantitative (velocity, mm s^−1^)	Brain tissue motion [63–65]	cardiac gating, flow-sensitive encoding, rebinning for cine SCIMI-MRI	no (research prototype) SCIMI MRI on GE systems
tagged MRI (GE, Siemens, Philips, Canon, Hitachi, Toshiba, United Imaging Healthcare)	tagged MRI	quantitative (if combined with post-processing such as harmonic phase (HARP) analysis)	brain tissue motion over the cardiac cycle [66,67]	cardiac gating (a real-time, research implementation has also been reported)	yes
analysis method based on the bSSFP sequences	amplified MRI (aMRI) or q-aMRI	qualitative (aMRI). Semi-quantitative (q-aMRI) (mm)	brain tissue, amplified tissue motion over the cardiac cycle and intracranial wall dynamics [ 8,9,18,68,69]	cardiac gating. Optional: respiratory gating	no (research prototype)
dynDWI (GE, Siemens, Philips, Canon)	dynamic DWI	quantitative (ADC)	CSF pulsations in the perivascular subarachnoid space [70–74]	cardiac/respiratory logging	on some vendors
fMRI (GE, Siemens, Philips, Canon, Hitachi, Toshiba, United Imaging Healthcare)	functional MRI (fMRI)	non-quantitative	brain tissue and CSF inflow at the fourth ventricle simultaneously with haemodynamic in the tissue [75–80]	optional: cardiac and respiratory logging	yes

### 2.1. Brain tissue

**Figure 1.**
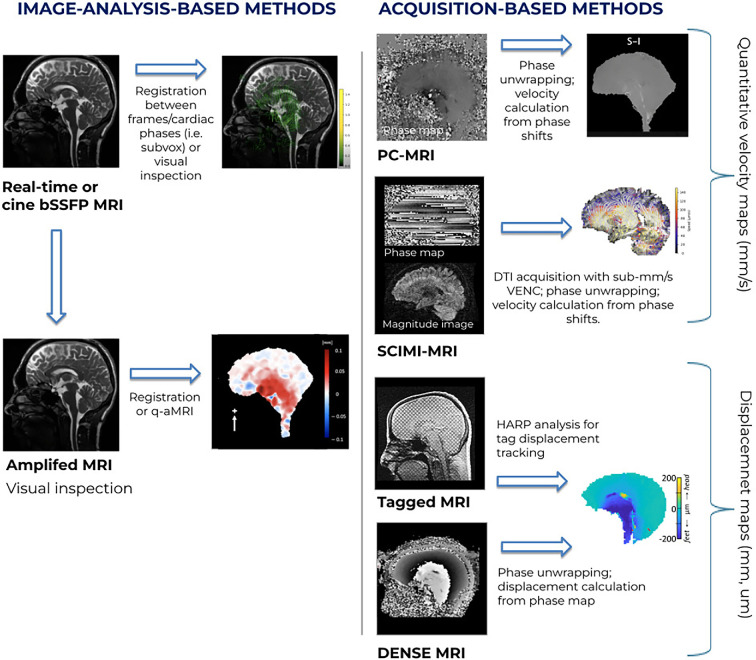
Graphical overview of MRI methods that are used to capture brain tissue motion. We distinguish between image-analysis-based methods and acquisition-based methods. Image-analysis-based methods aim to derive brain tissue motion from normal anatomical images that can in principle be acquired on any MRI scanner, whereas acquisition-based methods use dedicated MRI acquisition methods that are by design tuned to capture brain tissue motion and often need programming of the MRI acquisition software. To be able to use normal anatomical images for studying brain motion, the images are acquired as a time series, either synchronized to the heartbeat (‘cine’) or a real-time time series. Synchronization to the heartbeat often requires a special commercial software option, like the cardiac package on the MRI scanner, while real-time acquisitions require very fast imaging which is normally limited to a single slice. The output yielded by the various methods is indicated and varies from semi-quantitative displacement maps to truly quantitative maps of the displacement or velocity of the tissue, time-resolved over the cardiac cycle. It should be noted that both approaches can detect sub-voxel motions, i.e. displacements that are (much) smaller than the dimensions of a voxel (‘a 3D pixel’) in the image. Quantitative displacement or velocity maps can be used to subsequently compute tissue strains or strain rates by taking spatial derivatives.

A high-level summary of the main methods for capturing brain tissue pulsatility is provided in figure 1 with examples of use cases. The earliest MRI measurements of brain tissue pulsatility used ‘phase contrast velocity encoding’, known as PC-MRI [81–84]. PC-MRI makes use of motion-sensitizing magnetic field gradients, which induce phase shifts in the MRI signal that are proportional to the velocity of the tissue or fluid in the field of view. Using this method, tissue velocities of the order of 0.5 mm s^–1^ were identified [82], and Greitz *et al*. inferred brain tissue swelling during systole from observations of induced CSF velocities, such as the observed aqueductal flow, in relation to arterial inflow and venous drainage [83].

Various forms of PC-MRI have been used since then, with data quality improving alongside advancements in gradient performance and MRI technology [46,48]. These improvements enabled the estimation of brain tissue stiffness from the measured brain tissue pulsatile velocities [48] and the quantitative estimation of the volumetric strain across relatively large regions of interest in the brain [46]. A more recent innovation is the phase-sensitive reconstruction of diffusion tensor data (simultaneous coherent/incoherent motion imaging (SCIMI)), allowing the assessment of coherent brain motion with a velocity encoding value of 0.18 mm s^−1^ [85]. This approach has the benefit that it allows for simultaneous analysis of the diffusion tensor from the same data.

As the above-mentioned methods are based on velocity-induced PC, time integrals are required to estimate brain tissue motion or strain over the full cardiac cycle, making them prone to noise accumulation. It is therefore no surprise that alternative methods have been developed to directly measure tissue displacement over time.

Soellinger *et al*. were the first to show the ability of MRI tagging to measure brain tissue motion [67]. Originally developed for imaging heart motion, MRI tagging uses radio-frequency pulses and field gradients prior to the image acquisition (‘pre-pulses’) that create ‘tags’ by modifying the magnitude of the longitudinal magnetization (the basis of the MRI signal) of the tissue. A common tagging pattern is a sinusoidal variation of the magnetization with a given spatial frequency (or ‘tag spacing’), along a given direction in space. This pattern is subsequently visible in the images as dark lines that move together with the moving tissue.

One way to analyse this spatial tagging pattern is the harmonic phase (HARP) analysis method, which detects the local phase shifts of the tagging pattern relative to an undeformed reference pattern [67,86]. HARP applies a bandpass filter to the Fourier transform of the image, to isolate the spectral peak that corresponds to spatial frequency of the tagging pattern. The change over time in the phase of the resulting image (the ‘harmonic phase’) reflects the shift of the tagging pattern. Interestingly, this method is very similar to displacement encoding with stimulated echoes (DENSE). DENSE uses a stimulated echo with displacement-encoding gradients. The first two 90° pulses together with a displacement-encoding gradient create a sinusoidal variation in the longitudinal modulation, just as the pre-pulses in MRI tagging. The subsequent selective acquisition of the stimulated echo in DENSE can be seen as applying a bandpass filter during acquisition, something which HARP achieves by way of image post-processing [87].

**Figure 2.**
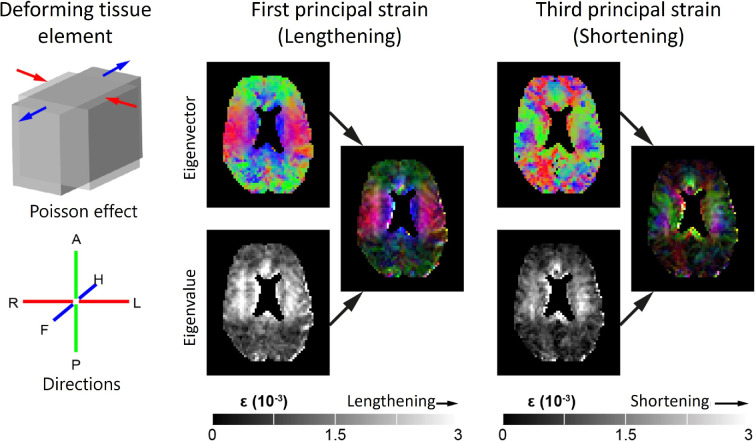
Brain tissue deformation as imaged by DENSE MRI. The maps represent the strain tensor that describes the tissue deformation induced by the heartbeat. Left, top: schematic depiction of a deformed voxel at peak systole relative to its shape at end-diastole. It illustrates how a voxel stretches along a certain one-dimensional direction (blue arrows), while at the same time, it shortens along another orthogonal direction (red arrows), which is known as the Poisson effect. Left, bottom: the RGB colour coding of directionality. Red: right to-left (RL); green: anterior-to-posterior (AP); and blue: feet-to-head (FH). Middle and right panels show the direction and magnitude of first principal strain (lengthening) and third principal strain (concomitant shortening perpendicular to the first principal strain) in each voxel for a healthy volunteer, respectively (unsmoothed data from DENSE measurements at 7 T MRI). Top: the direction of the eigenvector in each voxel using the directionally encoded colour scheme. Bottom: grey-scale maps representing the magnitude of the associated principal strains. For the third principal component, the absolute value of the eigenvalues was taken, resulting in a map with only positive values. Multiplying the eigenvectors with the eigenvalues results in an intensity colour map that summarizes both the direction and magnitude of the respective strain components. Figure adapted with permission from [91, fig. 2] (CC BY-NC-ND 4.0).

The major difference between HARP and DENSE is that DENSE allows one to choose motion-sensitizing (‘tagging’) gradients that yield a tag spacing well below the imaging resolution—something not possible for traditional tagging. Thus, DENSE allows for full optimization of the motion sensitivity up to the fundamental limits [88]. At this point, a further increase in the strength of the motion-sensitizing gradients to increase the sensitivity to coherent motion would be outweighed by the simultaneously increased signal loss due to diffusion or flow (which erase the spatial tagging pattern). DENSE has been shown to be very well suited for quantifying the subtle brain tissue motion both at 3 T MRI [89] and, more recently, at 7 T MRI [90]. At 7 T, an optimized DENSE acquisition allows for the assessment of the full strain tensor of brain tissue deformation induced by the heartbeat in three dimensions and time-resolved over the cardiac cycle. This is achieved with a spatial resolution of 3 mm isotropic and a temporal resolution of approximately 50 ms in 5 min [91]. An illustration is provided in figure 2.

Using DENSE, local volumetric strain in response to cardiac pulsations has been reported, with values of the order of 10^−4^. Grey matter volumetric strains appear to be significantly higher than white matter, which could be due to differences in blood volume (vascular density), intravascular pulse pressure or stiffness between these tissue types. These changes, which correspond to brain volume expansions of around 0.5 ml, are in good agreement with changes in CSF [92], implying that venous compression acts to maintain intracranial pressure near constant during the cardiac cycle. In a recent study, intracerebral compressions have been observed that are consistent with venous compressions [62].

bSSFP is an MRI technique that excels in brain imaging due to its high signal-to-noise ratio and rapid acquisition capabilities [93]. The efficiency of bSSFP comes from its rapid acquisition time, making it well suited for imaging dynamic processes within the body, including the brain. In brain imaging, bSSFP has been used to capture the motion of the brain tissue.

Cine bSSFP MRI techniques involve the sequential acquisition of images across multiple phases of the cardiac cycle, enabling the creation of time-resolved ‘cine loops’ that capture the cyclical motion of brain tissues and fluids. In other words, cine MRI effectively generates a ‘movie’ of brain motion throughout the cardiac cycle—that represents one cardiac beat. Note that this cine MRI approach is also used by PC-MRI methods. However, the cine bSSFP approach offers exquisite brain tissue contrast and has been shown valuable in visualizing the pulsatile motion of the brain tissue and the flow dynamics of CSF within the ventricular system. By providing high temporal resolution, cine MRI offers assessments of brain tissue biomechanics and has been helpful for analysing tissue deformation in applications such as idiopathic syringomyelia and Chiari malformation [6].

Real-time MRI offers continuous, non-gated imaging of the brain, allowing for the capture of spontaneous and rapid movements that may occur outside of the cardiac or respiratory cycles [76]. This technique is especially useful in clinical scenarios requiring immediate assessment of brain motion, such as intraoperative settings or in patients unable to remain motionless. Real-time MRI provides a dynamic view of brain tissue motion, capturing transient brain movements and offering a dynamic view of brain tissue mechanics.

However, both cine and real-time bSSFP methods rely on image analysis to derive brain tissue motion, which limits their ability to capture sub-voxel pulsatile motions, making it challenging to detect subtle brain movements. Attempts to extract the sub-voxel motion in cine bSSFP have been made by Laven *et al*. who used a phase-based registration approach [94] to highlight elevated cerebellar tonsil and brainstem motion in Chiari malformation patients [95]. In a way, the phase-based registration approach may be seen as a variant of the HARP method, but now using the intrinsic (though limited) contrast in the image as a tagging pattern.

Another approach to overcome these limitations is amplified MRI (aMRI) [69,96–99], which amplifies small tissue displacements by applying motion magnification algorithms such as Eulerian video magnification [100] and phase-based motion magnification [101] to the cine bSSFP MRI data. This technique amplifies otherwise imperceptible tissue deformations and fluid dynamics, providing promising insights into neurological conditions such as Chiari malformation [7], neurodegenerative diseases [18], hydrocephalus [8], acute intracerebral haemorrhage [25] and in assessing aneurysm wall stability through a variant known as aFlow [9,68].

More recently, quantitative aMRI (q-aMRI) has been introduced, which allows for the extraction of the displacement field, enabling the visualization and quantification of the sub-voxel pulsatile brain motion in physical units [102]. Simulations show that three-dimensional (3D) q-aMRI can accurately quantify sub-voxel motions as small as 0.01 times the voxel size. The coupling between brain tissue motion and CSF flow in the aqueduct has also been captured using PC-MRI and q-aMRI [102]. It was demonstrated that the flow/motion profile extracted from q-aMRI, which reflects tissue field displacement and ventricular shape changes, was comparable to the CSF flow profile measured by PC-MRI in the cerebral aqueduct, highlighting the dynamic interplay between brain tissue deformation and fluid motion.

While bSSFP-based MRI methods, including those using registration-based approaches and aMRI, are fast and provide exquisite tissue contrast, they do not directly measure displacement during the acquisition, unlike techniques such as PC-MRI, MRI tagging and DENSE.

### 2.2. Magnetic resonance elastography

Elastography is the science of non-invasively creating quantitative maps of the mechanical characteristics of tissues. It employs an imaging modality, such as ultrasound, MRI or optical imaging, to measure and map the distribution of internal tissue displacements that result from shear stress. The shear stress may be quasistatic or dynamic and be generated externally via a vibrating actuator, or be intrinsic to the tissue from cardiovascular pulsations, respiration, vocal cord vibrations, etc. The measured displacement fields can be used to reconstruct the tissue properties via various types of inversion methods, including algebraic inversion [103] and nonlinear viscoelastic inversion [104], which allow the mapping of the viscoelastic properties of soft biological tissue.

Magnetic resonance elastography (MRE) with extrinsic activation has been used to characterize mechanical properties *in vivo* [105–108]. MRE based on intrinsic actuation by the brain’s natural blood vessel pulsations has also been explored over the past decade [46,48,55]. Recently, using optimized brain pulsation measurement at 7 T MRI, MRE with intrinsic activation has been shown to provide detailed spatial information comparable to an atlas obtained with extrinsic activation [60,109]. In these MRE studies, brain tissue is mostly modelled as a plain, viscoelastic material. However, the mechanical behaviour of the human brain tissue is more complex and depends on many factors, including cell density [110], anisotropy [111], perfusion [112], pulsatility [113], myelination [114], inflammation [115], vascular density [116] and functional activation [117]. Advanced imaging techniques and more complex models such as poro(visco)elastic models will be required to identify the individual contributions of these factors [118].

### 2.3 Neurofluids

**Figure 3.**
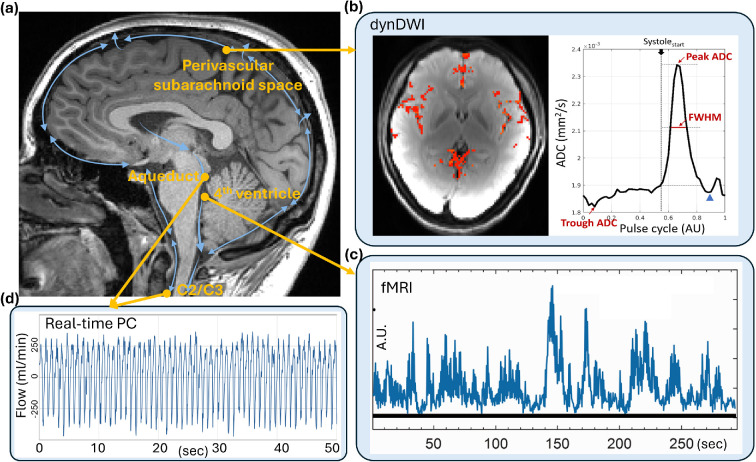
Illustrations of representative dynamic MRI techniques for capturing neurofluid pulsations, including dynamic diffusion-weighted imaging (dynDWI) for assessing the CSF pulsations in the perivascular subarachnoid space; functional MRI (fMRI) for capturing CSF inflow at the fourth ventricle; real-time and 4D PC-MRI for quantitatively measuring CSF flow at the C2/C3 level or the aqueduct; and balanced SSFP for semi-quantitatively measuring CSF flow along the ventricles and spinal canal. Abbreviations: ADC: apparent diffusion coefficient; FWHM: full width at half maximum; A.U.: arbitrary unit; sec: seconds; bSSFP: balanced steady-state free precession.

Dynamic MRI techniques can also be used to capture neurofluids’ pulsations. An illustration of the techniques presented in this section for capturing neurofluid pulsations is provided in figure 3.

For fluids with (largely) coherent flows, the PC methods mentioned above can be applied when appropriately tuned to typical fluid velocities by adjusting the strength of the motion-sensitizing gradients. Pulsatility in the brain’s major feeding arteries, draining veins and the CSF in the aqueduct and spinal canal have been extensively studied [12,17,47,49,119,120].

The velocity pulsatility in distal cerebral arteries (diameter 1 mm and above) is also within reach with state-of-the-art four-dimensional (4D) PC angiography methods [121]. 4D PC is also increasingly used for studying CSF dynamics [122]. With 7 T MRI, optimized two-dimensional (2D) velocity PC sequences can push the limit to smaller perforating arteries in the white matter, with diameters estimated between 50 and 80 μm or larger [43,123,124], and small cortical veins with diameters of 0.6 mm or larger [44]. Although partial volume effects lead to underestimation of the velocities at these tiny diameters, the pulsatility in these small perforating arteries can still be studied and has been observed to be increased in patients with cerebral small vessel disease [11] and in older individuals [125,126].

A further advancement is the use of real-time PC acquisitions, which allow for the study of dynamic effects like respiration and other low-frequency oscillations, such as those related to vasomotion and sleep [50,52,54].

Dynamic diffusion-weighted imaging (dynDWI) has been developed to measure cardiac pulsation-driven CSF dynamics in the subarachnoid space, as has been extensively reviewed by Wright *et al*. [127]. The CSF dynamics in this space appear to exhibit incoherent motion, likely due to laminar flow and/or the stirring effect caused by cardiac pulses in the presence of trabeculae [128]. To capture these dynamics throughout the cardiac cycle, motion-sensitive field gradients with a relatively weak diffusion weighting (*b*-value below 200 s mm^−^²) are applied. Both 3D turbo-spin-echo (TSE) [70–72] and 2D echo-planar imaging (EPI) [73,129] techniques have been developed for this purpose. With TSE approaches it is in principle easier to suppress blood signal contributions by applying long echo-times while maintaining the *b*-value relatively low (blood has a relatively short *T*2 value compared to CSF).

The 3D TSE method offers higher spatial resolution and better suppression of blood signals due to its longer echo time; however, its temporal resolution is limited by the need for multi-shot acquisitions, allowing only four to six cardiac phases to be resolved within a clinically feasible scan time. In contrast, the 2D EPI readout enables rapid whole-brain imaging with a repetition time of less than 2 s, capturing up to 50 cardiac phases in under 5 min [73,74]. This facilitates detailed waveform shape analysis and has been recently applied to study cardiac pulsations in the perivascular space of arterioles within the parenchyma [130]. In 2D EPI, blood signals are suppressed as the motion-sensitive gradients used for diffusion-weighting spoil the signal of the fast-flowing blood [131–133].

Both methods have demonstrated that CSF dynamics in the subarachnoid space are strongly coupled to cardiac pulsation [134], with less pronounced coupling to respiration. This suggests that subarachnoid paravascular CSF pulsations are distinct from other circulation pathways, such as the aqueduct, which is coupled to both pulsation and respiration. Additionally, the pulsatility of CSF has also been investigated using gated intravoxel incoherent motion (gated-IVIM) in the subarachnoid space and the ventricles [135,136].

The DENSE MRI sequence, used to study brain tissue pulsations as above, is essentially the same sequence as the stimulated echo acquisition mode (STEAM) sequence used for diffusion-weighted imaging. However, while DENSE primarily focuses on the phase of the signal, which reflects coherent motion, STEAM focuses on the magnitude of the signal, reflecting signal loss due to incoherent motion, such as diffusion. Consequently, the DENSE sequence can simultaneously provide both strain data and diffusion data in the brain when the magnitude data are also analysed. In a study involving healthy volunteers, variations in the apparent diffusion coefficient (ADC) over the cardiac cycle were found to correlate with brain tissue strain rate, suggesting that the heartbeat-related tissue strains induce motion in the ISF [137]. Of note, this study avoided contributions from microvascular blood, by ensuring a sufficiently high minimum diffusion weighting (*b*-value of at least 300 s mm^−^²).

A similar approach has also been applied to the more conventional pulsed gradient spin echo (Stesjkal–Tanner) diffusion-weighted imaging under the name SCIMI [63–65,85,138]. It should be noted that the coherent motion in these measurements likely reflects tissue motion, particularly at stronger diffusion weightings (high *b*-values), where signals from free fluid are largely suppressed.

Functional MRI (fMRI), traditionally used to study neuronal activity, has also been applied to investigate brain pulsations in various neurofluid pathways, including pulsatile blood flow in cerebral arteries and sinuses [139,140], as well as CSF dynamics in the fourth ventricle. The fMRI acquisitions often have a relatively short repetition time compared to the time needed for the signal to recover (T1 relaxation time). This means that the tissue signals are lower than signals from blood or CSF that are freshly flowing into the slice from outside the imaged volume. This means that the slices at the edge of the imaging volume can be used to study the dynamics of the inflowing fluids. With its high temporal resolution and sensitivity to both haemodynamics in the cortex and CSF flow at the fourth ventricle, fMRI is a powerful tool for simultaneously mapping these dynamics and exploring the coupling between haemodynamics and CSF, enhancing our understanding of the brain’s physiology [75,77,78,80,141].

Building on the same contrast mechanism as fMRI (T2*-weighted), ultra-fast magnetic resonance encephalography (MREG) uses highly under-sampled 3D acquisitions to achieve even higher temporal resolution. This method enhances the speed of imaging, reaching a temporal resolution of 10 Hz with a repetition time of 100 ms [76,79,142,143]. With this high temporal resolution and whole-brain coverage, MREG can effectively resolve the three major drivers of neurofluid dynamics in its power spectrum: cardiac (approx. 1 Hz), respiratory (approx. 0.3 Hz) and low- or very-low-frequency oscillations (0.001−0.1 Hz). This capability makes MREG a powerful tool for capturing spatio-temporal brain pulsations across different oscillation frequencies to understand neurofluid dynamics and brain pulsations. However, due to the T2* contrast mechanisms, both fMRI and MREG signals may be influenced by various signal sources, such as the blood oxygenation level-dependent effect, intravoxel changes in blood and CSF volumes and flow velocities, head motion and time-of-flight effects for inflowing fluids (blood and CSF). These overlapping signal sources can complicate result interpretation, particularly given the relatively large voxel size of 2.5−3.0 mm isotropic resolution.

Alternative approaches are based on MRI after the administration of contrast agents, either in the blood or in CSF, which both can shed light on different parts of the clearance pathways in the brain. We refer the reader to a recent and excellent review on this topic [144].

### 2.4 Future developments

In summary, this section has outlined advanced MRI techniques used to capture brain tissue motion and the dynamic fluids that influence its movement, focusing on both brain tissue and neurofluids. The review has highlighted acquisition-based methods such as PC-MRI and SCIMI, which provide quantifiable measures of velocity, as well as tagging techniques like tagged MRI and HARP, alongside DENSE, which generate displacement maps. Image-analysis-based methods using cine and real-time bSSFP have been employed to measure larger-scale changes in brain motion, while aMRI methods offer the ability to visualize and quantify more subtle, sub-voxel brain motions.

For neurofluids, which include blood, CSF and ISF, PC methods have been extensively used to study coherent flows such as the pulsatility of blood in arteries and veins and the movement of CSF in the aqueduct and spinal canal. Innovations such as dynDWI and real-time PC-MRI have advanced our ability to capture the dynamics of these fluids along various CSF circulation pathways. Techniques like q-aMRI have allowed for more precise mapping of the interplay between brain tissue deformation and fluid motion and fMRI for assessing the coupling between haemodynamic changes and CSF in-flow at the fourth ventricle. These approaches have shed light on the coupling between brain tissue motion and CSF flow, offering deeper insights into conditions such as idiopathic intracranial hypertension and hydrocephalus.

**Figure 4.**
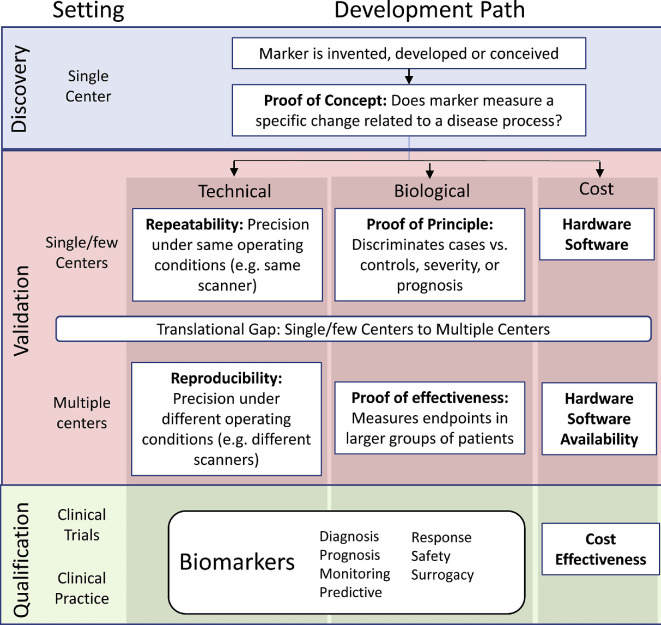
Suggested framework for the development of imaging biomarkers for cerebral small vessel disease, highlighting the unmet needs in measuring and characterizing brain pulsatility. Accurate and biologically specific methods are still required to address gaps in understanding capillary pulsatility, distinguishing fluid volume from flow velocity pulsations at the microvascular level and identifying the origin of fluids from arterial, venous or perivascular compartments. Figure reproduced from [145, fig. 1] (CC BY-NC-ND 4.0).

However, several unmet needs persist. Using the suggested framework for the development of imaging biomarkers, suggested for cerebral small vessel disease [145], there are still many aspects of the physiology of the pulsating brain that are in need of the development/discovery of accurate and biologically specific measurement methods (figure 4). To name a few: how can we measure and characterize capillary pulsatility? Velocity-selective arterial spin labelling techniques [56,146,147] hold potential but need further development and validation [57,58]. How can we distinguish fluid volume pulsation from fluid flow velocity pulsations at the microvascular level? Here, vascular space occupancy (VASO) MRI [148] has recently been proposed to provide further insights [59]. Can we further distinguish whether these fluids belong to the arterial, venous or perivascular/ISF compartments? Other methods that have shown proof of concept of targeting specific aspects of the pulsating brain are still in need of further technical validation before the translational gap to other centres and even the clinic can be made.

Finally, given the level of complexity of the pulsating brain physiology, it seems natural to couple advanced measurements with model-based image analysis. This will be discussed in §4.

## 3. Ultrasound studies

The development of ultrasound techniques provides the possibility to assess brain biomechanics and has several clinical advantages over other forms of imaging. Ultrasound is safe, portable, easily available and well tolerated by patients, making it exceptionally suitable for population screening, point-of-care and serial monitoring applications.

Unlike other imaging techniques, ultrasound involves a mechanical (longitudinal pressure) wave, and such waves are especially well suited for assessing fine tissue structure, density and bulk (compressional) elastic modulus. Vessel wall motion, soft tissue motion and blood flow can be visualized in real time using Doppler and speckle tracking techniques. In addition, ultrasound elastography provides images and regional estimates of tissue shear viscoelastic properties such as stiffness, which are key for modelling biomechanical properties. Overall, ultrasound is a very suitable approach for probing cerebrovascular haemodynamics, neurovascular coupling, tissue stiffness and motion and the interplay between them. However, the feasibility of using ultrasound to capture CSF and ISF circulation non-invasively appears to be largely unexplored.

Ultrasound imaging of the brain is already part of current clinical practice [149,150]. However, the presence of the skull is a limiting factor. This is because the skull reflects and absorbs the ultrasound beam, resulting in aberration and limited beam penetration. Hence, current clinical ultrasound imaging applications are mainly limited to brain imaging in neonates (through the fontanelle) and insonation through acoustic windows, such as the temporal (located above the zygomatic arch), submandibular, transorbital or occipital windows.

Ultrasound-based approaches exist for evaluating brain tissue motion but are still at a relatively early stage of development. The first approach is tissue pulsatility imaging (TPI), which captures motion within a 2D sector of brain tissue accessed through acoustic windows. The second one is transcranial tissue Doppler (TCTD), which addresses the limitations imposed by the skull by using low-frequency ultrasound, therefore enabling deeper penetration, and exploits the fact that Doppler information is largely preserved through the skull to detect tissue motion along a single one-dimensional (1D) beam line. These two approaches are introduced here, but the reader is referred to two recent review articles for more information [22,151]. Finally, the section introduces ultrasound elastography that may be used for estimating tissue viscoelastic parameters *in vivo*, used in brain mathematical models of pulsation, and, possibly, for capturing tissue motion.

### 3.1. Brain tissue pulsatility imaging

Imaging ultrasound systems in brightness mode (B-mode) can be used to visualize soft tissue and measure arterial and venous blood flow velocity through a combination of imaging and Doppler techniques. B-mode returns a grey-scale, real-time, image of ultrasound echo amplitudes, sometimes referred to as an echogram. In systems equipped with a phased-array transducer, commercially available for echocardiography applications, TPI has been developed to study ventricular motion in the heart. In brain TPI, these systems have been adapted to visualize and measure brain motion through suitable modifications of the signal processing technique. Kucewicz *et al*. [152,153] pioneered the brain TPI approach, demonstrating ultrasound’s capability to visualize brain motion in real time.

Phased-array echocardiography probes produce a ‘fan-shaped’ field of view, making them well suited for imaging through narrow acoustic bone windows. Designed for imaging deep structures like the heart, these low-frequency probes (<4 MHz) can penetrate up to 15 cm into the brain. Through the temporal bone window, the fan-shaped view captures the midline of the brain and both hemispheres at the level of the circle of Willis.

**Figure 5.**
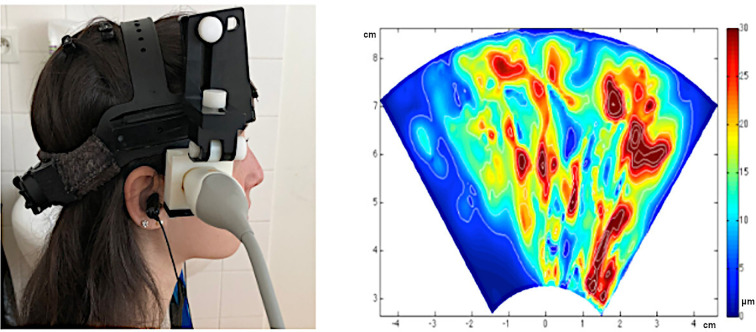
The typical set-up of the TPI equipment on a participant, with measurements taken from the right temporal position (left). The colour map shows the amplitude of brain tissue motion in the direction of the ultrasound beam with the brain moving inward (towards the midline) in systole (right). The map covers an axial slice of the brain centred on the Willis polygon (*x*-axis: posterior–anterior, *y*-axis: lateral–medial). The set-up shown in the figure was used in the M-Pulse study reported in [154].

Figure 5 illustrates a typical brain TPI set-up. Examples of clinically relevant investigations based on brain TPI include ageing [15], white matter hyperintensity [14] and depression [22,23].

### 3.2. Transcranial tissue Doppler

Since its introduction in 1982, transcranial Doppler (TCD) has been routinely used in clinical settings as a non-imaging diagnostic tool for measuring blood flow velocity in cerebral vessels and evaluating intracranial physiology and function [155,156]. Commercially available non-imaging TCD devices focus on estimating major cerebral artery blood flow for physiological monitoring of pathologies such as sickle cell disease [157], intra-operative embolus detection [158], stroke [159] and subarachnoid haemorrhage [160]. Non-imaging TCD is also used in clinical research to investigate cerebral blood flow regulation [13,161,162] and neurovascular coupling [163,164].

Continuous real-time measurement of brain tissue pulsatility using TCTD ultrasound was first demonstrated by Turner *et al*. [165] following modifications to existing TCD hardware.

Using TCTD, pulses of ultrasound are emitted from 2 MHz piezoelectric single-element transducers to depths of up to 80 mm below the probe’s surface. Tissue velocity is estimated perpendicular to the skin’s surface by processing backscattered ultrasound from tissue. One major advantage of TCTD is that measurements can be performed from any position on the skull, as tissue scattering is significantly stronger than blood scattering in conventional TCD, eliminating the need for acoustic windows.

Since the initial demonstration, a further experimental TCTD device has been developed, known as brain tissue velocimetry. The reader is referred to [151] for a review of developments, illustrations of the TCTD set-up, limitations and clinically relevant findings [166,167] obtained with these prototypes.

### 3.3. Ultrasound elastography

Ultrasound elastography methods provide an alternative source of *in vivo* brain tissue mechanical property data to those of MRE presented in §2. Ultrasound radiation force can be used to generate dynamic stress at a sound beam’s focus at depth in the tissue. The resulting tissue displacements, measured by using either Doppler or speckle tracking methods, may be displayed directly as a displacement image, converted to a strain (deformation) image or, if the stress was dynamic, used to estimate shear wave speed and attenuation for viscoelastic modulus imaging. The last of these methods is known as shear wave elastography (SWE). Reviews of ultrasound elastography technologies have been published (e.g. [168–173]). The methodology continues to evolve, even though diagnosticians have been using tissue stiffness by observing the displacements of ultrasound echoes resulting from pressing on soft parts of the body surface as long ago as the late 1970s/early 1980s [174–176].

Transcranial ultrasound elastography is difficult through mature skull bone, which greatly distorts and attenuates the acoustic waves. Most experience of ultrasound elastography of the brain has therefore been obtained intraoperatively, after craniectomy [177–180]. Surgeons already subjectively evaluate lesion stiffness and adherence to surrounding brain, to optimize and achieve maximal resection. Passive intraoperative displacement and strain imaging has been employed using intrinsic tissue pulsations [181,182]; tumours were sometimes seen as having a different stiffness from surrounding brain, and time sequences showed pulsations of displacement and strain at selected locations. In [183], strain imaging while continuously vibrating the ultrasound probe by 0.3 mm at 5−10 Hz demonstrated that tumours may have stiffness equal to, softer than or stiffer than surrounding brain and that tumour boundaries may exhibit high strain. Later, Prada *et al*. [184] found that strain images may provide sharper tumour margins than echograms. Chakraborty *et al*. [185,186] used freehand palpation with the probe, showing good agreement of strain contrast with surgical opinion of tumour consistency, and a high strain rim around some tumours at higher applied strain rates. The latter finding was taken to indicate low mechanical continuity with surrounding brain and had good correspondence with subsequent surgical opinion of ease of removal of the tumour. By imaging shear strain rather than normal strain, this finding was later developed as ‘slip elastography’ [187] to further characterize adherence between tumour and surrounding brain.

Features of strain images characteristic of non-adherent lesions were identified in intraoperative 3D strain images of brain tumours [182] and later validated by finite element modelling and phantom experiments [188]. Intraoperative SWE of the brain, using acoustic radiation force actuation, has also been employed [189–191], finding that (i) some epileptogenic lesions are detectable as stiff on elastography despite being MRI negative, (ii) variability exists across tumour types in tumour/brain stiffness ratio, (iii) measured tumour stiffness corresponds well with surgical opinion, and (iv) detection of residual tumour is most accurate when SWE is combined with surgical opinion. A multiparametric approach of echography with elastography, Doppler and/or contrast ultrasound has also been found valuable [192,193].

In the neonate, transfontanellar radiation force-based SWE has demonstrated that brain stiffness increases with preterm gestational age and that SWE could assist early identification of preterm babies at high risk of white matter damage [194]. In adults, ultrasound elastography through the temporal window, using external 60 Hz time-harmonic vibrations, has shown brain stiffness pulsations (of amplitude approx. 5% in the temporal tissue, approx. 11% in the basal cisterns and approx. 13% in the brain stem) that peak in synchrony with blood flow in the middle cerebral artery measured by ultrasound Doppler [195].

Confirmation that the MRE approaches presented in §2 and ultrasound elastography provide equivalent values is difficult, however, due to factors such as registration difficulties, time delay between measurements, the possible need to make the ultrasound measurements post-craniectomy (with consequent decompression), the effect of anisotropy in combination with differences in wave propagation direction and differences in the frequency and bandwidth of the shear waves. A rare attempt to compare measurements was made by Chan & Li [178,196], in post-mortem *in situ* brains of mice. When making such comparisons, it is important to note that it is typical in the MRE literature to provide values for the complex shear modulus G*, whereas in the ultrasound elastography literature, either shear wave speed is provided or the value of Young’s modulus, which has units of kPa as does the shear modulus.

### 3.4. Future directions

Ultrasound has substantial potential for advancing our understanding of brain motion in large cohort studies. Both brain TPI and TCTD are safe, low cost, easy to implement and well suited for such studies and for the clinical setting. Ultrasound techniques for measuring brain pulsatility are portable, non-invasive, repeatable, and bedside available, making them a potential routine tool. Notably, TCTD is also capable of providing real-time physiological measurements of brain tissue motion. The reader is referred to [22] and [151] for a discussion of expected future developments in brain TPI and TCTD, respectively. Objectives include standardizing acquisition and analysis methods for TPI and TCDT, building annotated databases to support large-scale studies and developing reference maps linking probe positions to brain anatomy. Extended pulsation waveform analysis, guided by principles similar to extensively used electrocardiogram and electroencephalogram libraries and possibly enhanced by machine learning, could inform diagnostic tools. Additionally, validating ultrasound recordings against established MRI techniques presented in the previous section will strengthen cross-modal reliability.

In the absence of the skull, ultrasound imaging would also be an ideal modality, offering high spatial resolution, good soft tissue discrimination and ultra-fast imaging capable of estimating tissue motion, blood flow and stiffness in real time.

**Figure 6.**
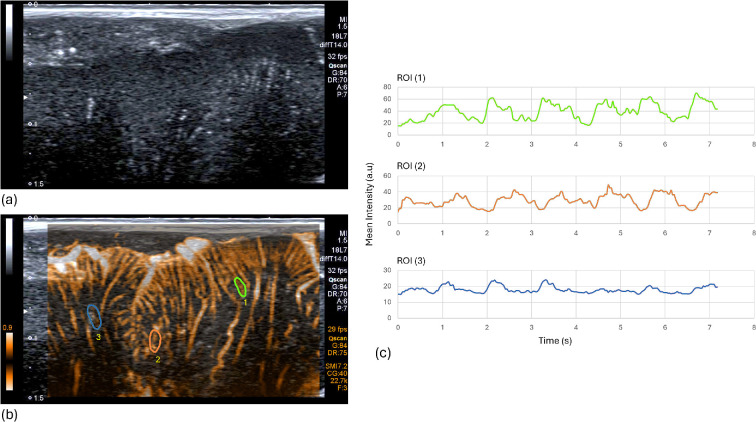
An example of some of the information that could be available with ultrasound imaging methods if it were not for the aberrating and attenuating effects of the skull, or if eventually full correction for these effects were to become possible. This example shows a high-frequency ultrasound B-mode image (*a*), time integrated microvascular colour power Doppler image retrospectively overlaid on the B-mode image (*b*) and three example time sequences of pulsations in Doppler power in small blood vessels (*c*) for the regions of interest (ROI) drawn in yellow in (*b*). Images (courtesy of Professor Christopher Uff) were acquired during routine recovery investigation of a decompressive craniectomy following trauma by scanning through the skin overlying the window where a small patch of skull had been removed. A Canon Toshiba Aplio 500™ was employed, with an 18L7 probe and Superb Microvascular Imaging (SMI)™ software.

For application in organs other than the brain, recent developments in Doppler signal processing have combined the strength, instantaneous Doppler shift and time-correlation of the echoes from blood to improve the discrimination between blood and tissue motion, resulting in a dramatic improvement in the resolution and flow-sensitivity of Doppler images (e.g. [197]). Such microvascular imaging modes have become commercially available and offer frame rates that allow blood flow pulsations to be observed. In the absence of the skull, such methods have the potential to provide some of the much-needed information, namely, to monitor and distinguish fluid volume pulsation from fluid flow velocity pulsations at the microvascular level. Imaging through the skin over a window in the skull created by decompressive craniectomy (figure 6) allows such advanced Doppler methods to be employed and is a technique that may provide opportunities for future basic research on the relationship between the spatial patterns of pulsatile microvascular blood flow properties and mechanical pulsations in the brain. In the breast, for example, microvascular Doppler has successfully been combined with ultrasound elastography (see above) for a more comprehensive microvascular/mechanical characterization of tumours [198].

In the presence of the skull, full-wave inversion imaging [199] could further advance ultrasound imaging of the brain, though this technology is not yet commercially available.

## 4. Modelling studies

Mathematical models can play a pivotal role in explaining the multiscale nature of biological phenomena, isolating and analysing physio-pathological processes, informing the design of experiments for data generation and ultimately interpreting complex datasets. Collaborative efforts—such as the Living Heart Project [4] and the Physiome Project [5]—have already exemplified how models ultimately help bridge gaps in empirical knowledge and support the development of improved diagnostic and therapeutic strategies. Hence, recent advances in the acquisition of brain pulsation data highlight the need for comprehensive mathematical models.

Mathematical models of the brain have a long history, although their application to simulate brain tissue pulsations is relatively limited. One reason for this is the complexity of coupling solid and fluid models together, which although performed in other organs has been limited in the brain. This is partly due to the very different lengths and time scales that are involved in brain function and development and partly due to the very limited amount of experimental data. We thus briefly review the models of brain tissue and fluid circulation, focusing on models derived from fundamental principles, before considering the coupling between these. In this review, solid mechanics (brain tissue) and fluid mechanics (blood, CSF and ISF) models are considered separately, for convenience, before the few models that explicitly consider the two together (‘coupled’ models) are described.

### 4.1. Solid mechanics

Mechanical factors play a key role in multiple different aspects of the brain’s function, with strong coupling between mechanical and electrophysiological behaviour [200]. Human brain tissue is ‘a porous, fluid-saturated, nonlinear solid with very small instantaneous volumetric compressibility and negatively charged molecules, capable of permanent deformations’ [200]. Despite, or perhaps because of, this, the mechanical properties of *in vivo* brain tissue remain very poorly characterized. This is since *ex vivo* measurements often provide only a poor estimate of the *in vivo* properties and because of the substantial difficulties in interrogating an organ that is so well protected mechanically.

Brain tissue is both ultrasoft and biphasic, making it difficult to control its deformation during testing. There is no agreed testing protocol for different lengths and time scales, and, likely as a result, values of brain tissue stiffness have varied significantly [201,202]. Studies using multiple mechanical tests have shown that brain tissue exhibits a distinct asymmetry between compression and tension and that there is a regional but not a significant directional dependence, with an isotropic modified Ogden model appearing to be a good fit [203,204]. Despite these recent studies, there remains a poor consensus on the values of the parameters for the mechanical properties of brain tissue.

Although brain tissue is highly nonlinear, the usual approximation of a linear relationship between stress and strain is a reasonable one for the small displacements encountered in tissue pulsations. In such a linear model, and neglecting viscoelastic losses, only two parameters need to be specified, typically Young’s modulus and Poisson ratio, with all other parameters being computed from these (e.g. [205]). Brain stiffness is regularly characterized by a value of Young’s modulus, which was proposed to be 584 Pa by Taylor & Miller [206], and this value has been used as a standard value by multiple authors since (alongside a value of Poisson’s ratio of 0.35), despite the fact that this is a much lower value than was previously used by earlier authors (around 3 kPa). Likewise, a permeability value of 1.4 × 10^–14^ m^2^ was proposed by Taylor & Miller [206], based on calculations by Kaczmarek *et al*. [207], and this has again been widely adopted in modelling studies. The study by Smillie *et al*. [208] gives a particularly good description of the choice of model parameters, based on this evidence. The studies by Tully & Ventikos [209], Chou *et al*. [210], Vardakis *et al.* [211], Guo *et al*. [212] and Vardakis *et al*. [213] then all use these parameter values as their baseline (with some amendments when proposing multiple compartments) to simulate the displacement of tissue in pathological conditions.

Other studies have, however, reported much larger values for Young’s modulus and Poisson’s ratio. The recent comprehensive survey by Morin *et al*. [214] tabulates around 30 studies (dating from 1999 to 2016), highlighting that brain tissue is quasi-incompressible (i.e. Poisson’s ratio close to 0.45), with shear modulus at small deformations of ‘about [a] few kPa’ and shear modulus differences between grey and white matter. It should be noted that the methods used to obtain these values are highly heterogeneous, but that the values obtained are quite significantly higher than for the other studies mentioned above. There is a large difference between the drained and undrained values of Young’s modulus, as discussed in detail in [208], with the drained values being much smaller than the undrained values (typically of one order of magnitude), which likely explains the discrepancy (but which again makes comparisons between studies highly challenging). The variability between studies is further amplified by the fact that a fluid-filled porous structure like the brain exhibits strongly frequency-dependent behaviour [215,216]. There are also significant increases in stiffness with ageing [217].

It is also worth noting that the literature on the viscoelastic properties of brain tissue is considerably sparser, and whether viscoelastic behaviour is relevant at the cardiac pulsation frequency remains to be determined. Grey matter and white matter have different viscoelastic properties, in terms of both stiffness and viscous time constants [203,204]. The multiscale properties of brain tissue have yet to be fully exploited in developing mathematical models, with only preliminary attempts to use the microstructure composition as a foundation for the macrostructure properties [218]. A more significant limitation is normally the simplified geometries that are assumed, and no study has yet considered the impact of changes in tissue geometry on the pulsations.

Computational models have also been constructed based on structural imaging data, such as MRI, in both human and animal models, with recent models including both grey and white matter as well as CSF and the skull [219]. These can be used in a similar manner to models of cerebral blood flow, described below, to simulate the response to stimuli such as post-ischaemic stroke oedema, with the potential for application to brain tissue pulsations in a fully 3D model, when coupled with models of fluid flow.

### 4.2. Fluid mechanics

Blood flows through the cerebral vasculature, which is a highly complex, structurally heterogeneous, inter-connected network of blood vessels that adapt over short and long time scales to provide a continuous supply of oxygen and glucose to brain tissue. Cerebral blood flow can be modelled at many different length scales, from the microcirculation (vessel diameter around 10 μm) to the large vessels in the circle of Willis (vessel diameter of a few millimetres). Models thus tend to take one of a number of forms, often described by the number of dimensions involved, from 0D models that have no spatial information to 1D models, with axial information along blood vessels, to full 3D models of the whole human brain. For a detailed recent review of all the different types of haemodynamics models, see [220].

The cerebral vasculature is highly responsive to physiological stimuli, matching cerebral blood flow to metabolic demand at both global and local scales, through a number of mechanisms known as cerebral autoregulation, cerebrovascular reactivity, neurovascular coupling and sympathetic activation [221]. In this context, the key parameter is permeability, which relates the flow through a volume to the driving pressure gradient. This can be estimated from detailed models of the vasculature (e.g. [222]) in the microcirculation, although in active systems this will not be invariant with time.

In addition to blood flow, the circulation of CSF and ISF plays an important role in the pulsatile flow field (see the reviews by Linninger *et al*. [223] and Kelley & Thomas [224]). This is particularly important due to the (limited) compliance provided by the spinal canal.

Models of blood flow and CSF first date from the study by Ursino [225], following which a number of electrical equivalent circuit models were proposed; see also early work by Stevens [226]. These are extremely simple to implement, being based on a handful of ordinary differential equations, but are compartmental models with no spatial information. 1D models have been used to simulate the pulsatile nature of blood flow and CSF, but these have yet to be coupled with tissue models and again do not provide full 3D information. The values of specific storage and permeability are rarely discussed in the literature. A recent study by Józsa *et al*. [227], based on optimization of a whole-brain model, yielded an arterial permeability value in the cortex of 1.234 × 10^–9^ m^3^ s kg^−1^, which is somewhat different from that used by other modelling studies (3.75 × 10^–8^ m^3^ s kg^−1^).

The simulation of brain pulsations also permits the quantification of enhancement to solute transport. There is an inertial steady streaming effect in the spinal canal, due to the coupling between advection and diffusion, which is similar to Taylor dispersion [228–230]. This could potentially play an important role in the transport of solutes, although the importance of this is still somewhat controversial. Studies in mice models have recently shown enhanced diffusion in the perivascular space for cardiac and low-frequency pulsations [36,40]. However, these models have yet to be coupled with a tissue model, the effects of which will need to be quantified more accurately.

Full-brain models have been constructed based on the mouse brain [231,232], based on advanced optical imaging methodologies and based on regions of the human brain [233]. Whole human brain models cannot be simulated due to the number of vessels, but homogenization methods have enabled the microvascular properties to be ‘coded’ within the macroscale properties [222,234], enabling whole brain simulations to be performed [227]. These models have thus now reached the stage that they can be applied for simulating brain tissue pulsations.

### 4.3. Coupled models

Due to the presence of the rigid skull, there is a strong coupling between changes in blood volume and changes in tissue volume, as per the Monroe–Kellie doctrine. Hence fluid–structure interaction is a key component of brain tissue pulsations. There are a number of methodologies that can be applied, based on the work described above, to simulate such pulsations. The most promising approach is the use of a poroelastic framework, where a solid matrix is coupled with a number of fluid compartments (typically three), which enables a full 3D model to be constructed with both solid and fluid pulsatility included. Some of these models have been based on brain pulsation data, including two-photon imaging for animals [36], PC-MRI in humans [235] and dynamic measurements of ICP.

Only a few studies have developed the computational modelling framework for pulsatile flow in the human brain; see, for example, the study by Causemann *et al*. [236], which uses a finite element model based on MRI data, with a linear elastic tissue model and an extracellular fluid network. However, as yet there have been very few models that are fully coupled fluid–structure interaction models, apart from a few preliminary studies that have explored specific pathological conditions, including haemorrhagic transformation [237] and oedema [24].

### 4.4. Future directions

There has been a great deal of work to simulate the brain either as a solid phase or as a fluid phase, although there is still little work considering this as a coupled fluid–structure problem. As a result, models have as yet made only limited contributions to the study of brain tissue pulsations. The computational cost of performing dynamic simulations in a full 3D geometry is a significant challenge, with many model parameters being poorly quantified, and the resolution of MRI scans reduces the detail of both the brain geometry and the vasculature, potentially introducing very significant errors.

Recent work into model estimation using neural networks has shown some preliminary success (e.g. [238,239]), and this is a key area for future research. Large uncertainties in parameter values can be addressed through Bayesian methods, which have undergone significant development for estimating dynamical systems [240].

It should always be remembered that the system is highly multiscale, meaning that studies are required into both the microstructure and the macrostructure. This is the case both for the solid matrix, where mechanical properties at the macroscale are governed by the behaviour at the microscale, and for the fluid compartments, where blood flow occurs over many different orders of magnitude. A suitable mathematical framework is needed to couple together behaviours across these different length scales at this particular, cardiac, frequency band. Some preliminary work has been performed here, but this is an area open for future development, to incorporate information across multiple length scales within a single model. This will also permit simplified models that can be solved at relatively low computational cost, given the restricted frequency band of interest.

In the future, it should be possible to explore the behaviour of the current simplified models within more complex geometries and to explore potential extensions to these models within a virtual population of representative subjects. This will exploit recent advances in MRI accuracy, as described elsewhere in this review. As experimental data are more closely linked to modelling simulations, it will then be important to consider the effects of other systemic variables, such as end-tidal CO_2_ and respiration patterns, on model simulations to explore more fully the multivariate nature of the human brain and ensure that modelling of brain tissue pulsations gives repeatable, robust and reliable results.

## 5. Conclusions

This perspective paper has highlighted current research in MRI, ultrasound and mathematical modelling to enhance our understanding of brain tissue motion and neurofluid dynamics. Each discipline offers unique contributions: MRI provides high-resolution imaging that can reveal subtle interactions between tissue and fluid; ultrasound offers the advantages of portability, cost-efficiency and real-time imaging, making it ideal for large-scale studies; mathematical modelling offers a framework to address the complexity of tissue and fluid interaction in the intracranial environment and physiology. Modelling advances will enable the simulation of virtual populations, offering opportunities to optimize diagnoses and treatments.

One technology excluded from the scope of this review is near-infrared spectroscopy (NIRS). Although it does not provide direct measurements of motion, NIRS is a well-established non-invasive method providing insights into brain function and haemodynamics, which will provide a complementary perspective in future research (e.g. [241,242]).

Collaboration between technical and clinical practice is essential. For example, validating ultrasound results with MRI may potentially enhance diagnostic precision and reliability. Establishing shared datasets and standardizing data collection and analysis methods can support diagnostic tools and support model development. While each of the previous sections has illustrated specific future objectives, a cooperative effort will ultimately lead to innovations in diagnosis and patient ‘care.

## Data Availability

This article has no additional data.
